# Despite COVID: showcasing new research in evolutionary biology from academic care-givers in the middle of a pandemic

**DOI:** 10.1098/rspb.2022.2131

**Published:** 2022-12-07

**Authors:** Loeske E. B. Kruuk, Sarah F. Brosnan, Maurine Neiman

**Affiliations:** ^1^ Institute of Ecology and Evolution, School of Biological Sciences, University of Edinburgh, Edinburgh EH9 3FL, UK; ^2^ Departments of Psychology and Philosophy, Neuroscience Institute, Center for Behavioral Neuroscience, Georgia State University, PO Box 5010, Atlanta, GA 30302-5010, USA; ^3^ Department of Biology and Department of Gender, Women's, and Sexuality Studies, Provost Faculty Fellow for Diversity, Equity, and Inclusion, University of Iowa, Iowa City, IA 52242, USA

**Keywords:** COVID-19, pandemic, care-givers, academic careers, mothers

The COVID-19 pandemic caused unprecedented upheaval to research programmes and academic careers worldwide. In particular, lockdowns and working from home are clearly documented to have adversely affected the careers of those with caring responsibilities (e.g. [1]). Parents who had to combine childcare and home-schooling while maintaining academic work were especially likely to have been negatively impacted, and there is strong evidence that such disruption—like so many impacts of childcare [2]—disproportionately affected mothers (e.g. [3]). Care-giving-related disruption is also particularly likely to have been damaging for early- or mid-career researchers, with those without tenure being most at risk of long-term consequences (e.g. [4]). Inspired by suggestions of ways that the academy might seek to address some of these problems [5], *Proceedings B* has compiled a Special Feature showcasing research that has been maintained despite such challenges, recognizing the impact of the pandemic on care-givers, with a particular but not exclusive focus on mothers. This Special Feature aims to highlight new research in evolutionary biology, broadly construed, with an emphasis on new questions and emerging approaches. In this introductory article, we summarize its compilation.

We solicited proposals showcasing research on fundamental principles and processes within evolutionary biology (the area of biology in which we, the editors of the Special Feature, work). Our main criterion was that first authors should be early- to mid-career (similar to pre-tenure in the typical United States system) and have provided care to young children or had equivalent substantial care-giving responsibilities during the pandemic and the associated lockdowns. We chose to focus primarily on researchers who identify primarily as women^1^, in the light of multiple lines of evidence indicating that the pandemic more severely impacted women than men [5–7] and given other long-standing gender inequities in academia (e.g. [2,8–10]). We were nevertheless also open to those with other identities who have been impacted by care-giving responsibilities, recognizing that many people outside of the ‘mother’ role were also substantially affected. We also wanted to encourage geographical diversity of the submitting authors, with the hope of promoting work from countries that were especially affected by the pandemic or that often lack adequate representation in scientific literature. In particular, we wanted to include submissions from authors from countries other than those in western Europe and North America, not least because *Proceedings B* itself currently suffers from a significant Western bias both in publications (e.g. [11]) and in editorial board membership, which it is working to overcome. We are conscious that as societies progress to ‘living with COVID’, lockdowns will become a memory that many of us would rather forget—and that for many more established researchers, disruption over a year or so may not be a major issue in the long term. We therefore hope that this Special Feature can serve as a reminder of the challenges generated by the pandemic and the lockdowns, and that such reminders might help improve policy when the next global crisis comes around.

Although most people accept that those with care-giving responsibilities faced additional pressures during COVID, we anticipated some ‘what about men?’ pushback to our focus on women. In actuality, we received markedly little of this reaction, especially relative to the substantial positive reception for the Special Feature evident on social media and via direct communications. However, we appreciate that there may have been concerns on this point. We want to emphasize that we are not in any way downplaying the impact that pandemic caring responsibilities will have had on male care-givers, especially those at early career stages. Indeed, an equivalent issue focussing on male care-givers could be an excellent complement to the issue featured here. The Special Feature was not restricted exclusively to mothers or females, or care-givers who identify as women, and we also received proposals from men, some of which are included in the published papers. Perhaps the best response to the concern of ‘what about men?’ is to point out that we also focused on early-career researchers, so we are similarly guilty of apparently ‘unfair’ exclusion of later career researchers. In both cases, we wanted to support a demographic that was, on average, more severely affected than others; later-career researchers, such as ourselves, are in an entirely different situation with regard to job stability and security. No-one has questioned the early-career focus.

The large response to the call for proposals was exciting for us, though the authors' accounts of the pandemic's impact in heightening the already-extreme job insecurity of academia made for dismal reading. We were humbled by the stories of perseverance and persistence described by the authors. A common theme that emerged related to difficulties encountered at points at which people were aiming for transition to the next stage, such as from PhD to postdoctoral, or postdoctoral to independent research fellowships; another was of the difficulties associated with caring for more than one child, or the absence of support owing to demands on partners' careers or distance from wider family networks. At some point, there will hopefully be full evaluations of the respective impacts of gender, career stage, geographical location, number of children (or other dependents), partner's career, proximity to wider family, and other relevant factors, on research productivity through the pandemic years of (at least) 2020–2022—which will be a substantial, and probably disheartening, task. We also hope that the experience of the pandemic may remind us to be more thoughtful and compassionate towards colleagues during even ‘routine’ challenges, such as caring for sick or ageing family members or other traumas, particularly for those without the protection of tenure or a permanent position.

In an ideal world, we would have published the vast majority of papers proposed for the Special Feature. However given space constraints, we could only invite a small subset of proposed papers for a full review. As with all submissions to the journal, some were accepted relatively swiftly, others required one or more major rounds of revision, and unfortunately, some were rejected, although we hope the review process provided useful feedback. A handful of the papers we invited based on the proposal were never submitted as full manuscripts, no doubt in part a reflection of the uncertainty and time pressures faced by authors. The accepted papers have been published online as they were accepted over the year and are now being published together as a single print edition. Ultimately, we are pleased to have an edition of *Proceedings B* with a heavily woman-biased ratio of early-career first authors. However, regretfully, our aim to increase geographical diversity was less successful than we had hoped, with only five of the final 18 papers coming from outside North America or Europe. This outcome was disappointing, and we hope to explore the broader associated issues relating to it in more depth in the future.

The Special Feature contains a series of papers featuring a mix of review articles and primary research. Some of these relate directly to the pandemic: Warrington *et al*. [12] explore the impact of pandemic changes in human activity on bird behaviour and find rapid plastic responses to the novel environment created by the pandemic. Zhao *et al*. [13] provide a new perspective on mutational and selection processes in SARS-CoV-2 by describing strand-specific mutation spectra and painting an initial picture of RNA editing on both genomic strands. Frank *et al*.'s comparisons of patterns of molecular evolution in angiotensin-converting enzyme 2, made famous as the host protein bound by SARS-family viruses, demonstrate evidence for strong selection pressure on these enzymes in bats relative to other mammals; their results provide deeper understanding of why some coronaviruses probably strike some mammal taxa with greater severity than others [14].

The non-pandemic papers cover a range of evolutionary topics. Onstein *et al*. [15] explore the botanical consequences of the 25 Myr mega-herbivore gap that followed the extinction of the non-avian dinosaurs 66 Ma, including speciation slowdowns and the loss of defence traits. Das & Ratnam [16] show phylogenetic overdispersion among evergreen tree metacommunities in a tropical montane zone of the Western Ghats, India, owing to unexpected persistence of tropical lineages at high elevations when large patch areas generate suitable microclimates. Lalonde & Marcus [17] use a combined mitogenomic and nuclear marker approach to reveal a complex story of reticulate evolution and reconstruct the dispersal and invasion history of the nymphalid butterfly genus *Junonia*, renowned for its ability to spread worldwide. Considering life-history evolution, Matesanz *et al*. [18] show how inherited negative effects of parental stress may override adaptive responses to drought in a wild lupin, questioning the adaptive value of transgenerational plasticity. Ahlawat *et al*.'s experimental evolution experiment [19] comparing host–parasite coevolution to host-only evolution in a fruit fly-bacterial pathogen situation revealed no evidence for different costs of post-infection survivorship between coevolving and non-coevolving hosts, hinting that the increased host immunity and pathogen virulence that can result from host–parasite coevolution might not confer major costs. Reviewing the current state of the field of the mechanisms underlying male-killing mediated by bacterial endosymbionts, Hornett *et al*. [20] provide a novel argument that selection imposed by male-killing and favouring host nuclear suppressors will often involve the evolution of host sex determination pathways.

In contrast with concerns about under-representation of females as authors of scientific papers, Archer *et al.* [21]'s meta-analysis quantifies the over-representation of females as study subjects in life-history studies, especially among birds and mammals where information on females is almost double that of males. Evans *et al*. [22] use a five-decade ‘chronosequence’ of guppies transplanted from high-predation to low-predation environments to demonstrate evidence for rapid parallel evolution in microbiome composition in response to the change in environmental conditions. Walsman *et al*. [23] use an eco-evolutionary model, inspired by a guppy system, to explore how behavioural responses of prey to predation risk may lead to increased prevalence and virulence of parasites owing to changes in grouping behaviour. Kanwal & Gardner [24] modelled the role of population viscosity on kin selection, finding that if dispersal is conditional upon local density, individuals in more altruistic neighbourhoods disperse more frequently and kin competition is relaxed, leading to a negative correlation between dispersal and altruism. Reyes *et al*. [25] tested the laboratory finding that brain size correlates with sexual traits in 18 wild populations of guppies from diverse habitats and found significant variation in relative brain weight and brain region volume across populations related to environmental conditions. Córdova-García *et al*. [26] found that seminal fluid proteins in an agricultural pest, the Mexican fruit fly, regulate female olfactory responses and memory formation, which could both suggest novel evolutionary functions of seminal fluid proteins and guide future control efforts.

Traditionally the focus of male–male interactions has been on competition, but Saldaña-Sánchez *et al*. [27] show that male spider monkeys' behaviour is influenced by some socio-ecological factors, such as food availability and proximity to the home range boundary, but not others, such as the presence of receptive females, suggesting ways that males in this male-philopatric, fission–fusion society can flexibly adjust their relationships to allow for both cooperation and competition. Lew-Levy *et al*. [28] found that information transmission about spear hunting in BaYaka foragers occurred through costly teaching, typically direct instruction, which differs from other domains in which learning occurs through lower-cost mechanisms. McCullagh *et al*. [29] studied the development of the auditory brainstem in naked mole rats, which diverge from other rodents in many physiological aspects owing to their highly specialized underground lifestyle; they show that despite this divergence, they have similar hearing onset as other rodents, although they show developmental differences in other respects. Finally, the collection includes studies of scientific practice itself, with Roche *et al*. [30] demonstrating slow improvement over the last seven years in the completeness and reusability of publicly available datasets associated with scientific publications in evolutionary and ecology, as well as a major effect of principal investigator on a publication on both metrics. In summary, we hope the diversity reflects *Proceeding B*'s remit to publish novel science of general interest covering the breadth of the biological sciences.

The COVID-19 pandemic has highlighted the challenges facing care-givers of all types, and in particular those without access to reliable childcare, from non-Western or non-English-speaking countries or smaller institutions, or without access to often implicit standards of formal training and informal upbringing in the unspoken norms of science and publishing. These issues are systemic, extending far beyond one pandemic, and have shone a harsh light on the suite of challenges that need to be addressed if we are to achieve what we believe is the widely held goal of an equitable, diverse and inclusive scientific community. However, it seems that discussions of diversity-related goals do still elicit the question: ‘Why will diversity produce better science?’ We briefly summarize here some thoughts on this very complex landscape. Paramount is the ethical and moral responsibility to remove the prejudices and barriers faced disproportionately by some and not others in any workplace. In addition, if ‘quality’ of science is the main concern, then at least three further points follow. Widening the pool of competitive talent from which to draw researchers could increase quality by force of numbers [31]. Further, science requires not just blue-skies thinking, but also application, communication and training [32,33]—all of which rely on connection with as broad an audience as possible. Also finally, a diversity of experience and outlook may make headway where well-trodden paths have not [32] (think of the impact of inter-disciplinary research on scientific progress). A recent relevant example is provided by Yang *et al*. [34], who show that gender-diverse teams produce papers that are more highly cited and more novel relative to papers authored by all men or all women. We hope that this Special Feature makes a useful statement of concern about inequities in academia that have been magnified by the pandemic and thereby helps establish a framework for future proactive steps aimed at countering these persistent challenges.

## Data Availability

This article has no additional data.

## Endnote

1 We use the term ‘women’ as a mechanism of inclusion, but recognize that there is a dynamic discussion around which words and spellings are the most inclusive.
